# 3D printable tough silicone double networks

**DOI:** 10.1038/s41467-020-17816-y

**Published:** 2020-08-10

**Authors:** Thomas J. Wallin, Leif-Erik Simonsen, Wenyang Pan, Kaiyang Wang, Emmanuel Giannelis, Robert F. Shepherd, Yiğit Mengüç

**Affiliations:** 1 Facebook Reality Labs, Redmond, WA USA 98052; 2 Department of Materials Science and Engineering, Cornell University, Ithaca, NY 14850 USA; 3 Sibley School of Mechanical and Aerospace Engineering, Cornell University, Ithaca, NY 14850 USA

**Keywords:** Materials for devices, Actuators, Polymers

## Abstract

Additive manufacturing permits innovative soft device architectures with micron resolution. The processing requirements, however, restrict the available materials, and joining chemically dissimilar components remains a challenge. Here we report silicone double networks (SilDNs) that participate in orthogonal crosslinking mechanisms—photocurable thiol-ene reactions and condensation reactions—to exercise independent control over both the shape forming process (3D printing) and final mechanical properties. SilDNs simultaneously possess low elastic modulus (*E*_100%_ < 700kPa) as well as large ultimate strains (d*L/L*_0_ up to ~ 400 %), toughnesses (*U* ~ 1.4 MJ·m^−3^), and strengths (*σ* ~ 1 MPa). Importantly, the latent condensation reaction permits cohesive bonding of printed objects to dissimilar substrates with modulus gradients that span more than seven orders of magnitude. We demonstrate soft devices relevant to a broad range of disciplines: models that simulate the geometries and mechanical properties of soft tissue systems and multimaterial assemblies for next generation wearable devices and robotics.

## Abstract (web summary)

Additive manufacturing processing requirements pose restrictions on materials and joining chemically dissimilar components. Here the authors use silicone double networks that participate in orthogonal crosslinking mechanisms for independent control of the shape forming process and final mechanical properties.

## Introduction

There are inherent advantages to devices constructed, even partially, from soft matter. Biological systems employ soft (i.e., Young’s Modulus, *E* < 10^8^ Pa) tissues for load management, shock absorbency, conformal contact for manipulation, and passive energy recapture, among other functions^1^. In robotics, the combination of low moduli and large extensibility permit versatile devices that can reversibly exhibit a wide, continuous range of deformation states^2^. In these synthetic systems, crosslinked poly(dimethylsiloxanes), commonly referred to as silicone rubbers, are ideal building materials due to their excellent mechanical properties, thermal resistance, and chemical inertness. This phenomenal performance originates from the highly flexible siloxane (Si-O-Si) backbone that imparts a high degree of molecular mobility and glass transition temperatures among the lowest found in common polymers (*T*_g_ < −100 °C)^2^. Owing to the industrial relevance of molding such elastomeric polymers (elastomers), there exist numerous commercial room-temperature vulcanizing (RTV) silicones with impressive elastomeric properties (*E* < 1 MPa, d*L/L*_0_ > 200%). However, conventional fabrication strategies (replica molding and injection molding) only directly yield simple, prismatic shapes.

Recent works employed commercial liquid silicone rubber materials as inks for extrusion-based three-dimensional (3D) printing to obtain more complex, unmoldable geometries^3–6^. However, the gelation kinetics and rheological properties of these inks limit the print fidelity for both high aspect ratio structures and overhanging features, as the deposited material spreads as it wets the substrate, or slumps prior to curing.^7^ Modifications to the chemistry that alter viscosity and curing rates fundamentally change the volumetric crosslink density of the material^3,5^, often to the detriment of the base elastomer’s performance. Nascent technologies based on kinetically trapping commercial resins as embedded ink within a viscous matrix offer the potential to print designs of greater complexity^8,9^. However, the viscous matrix complicates path planning and the endemic trade-off in resolution and print speed (i.e., higher resolution requires smaller nozzle diameters, which restrict the rate of material deposition) persists for many embodiments of this technology^2^.

By comparison, vat polymerization techniques such as stereolithography (SLA) photopolymerize objects from within liquid resins at rapid deposition rates (>10^6^ mm^3^ h^−1^)^10^. Building within liquid media provides buoyant support that enables the direct printing of high aspect ratio, hollow structures possessing features on the order of 100 μm^2^. A few studies used SLA to produce silicones (polysiloxanes) from the free-radical-initiated photopolymerization of custom formulations^11–14^. Yet, none of these materials possess the combination of desirable mechanical properties found in RTV silicones (low modulus, as well as high elongation and toughness).The processing requirements of SLA—rapid photopolymerization from a stable, low-viscosity (*η* < 5 Pa s) resin—precludes conventional strategies for improving the mechanical robustness of these materials^15^. For example, many commercial silicones incorporate particles, such as fumed silica, to improve both the ultimate elongation and toughness, but the volumetric loading fractions (*ϕ*_vol_ ~ 30%) necessary to see such significant improvements correspond to an increased viscosity well beyond the printable regime^16^. In addition, the solid particles stiffen the composite, hindering applications that require low moduli base polymers. Thus, SLA printing a single silicone network with ideal elastomeric performance remains elusive.

**Fig. 1:**
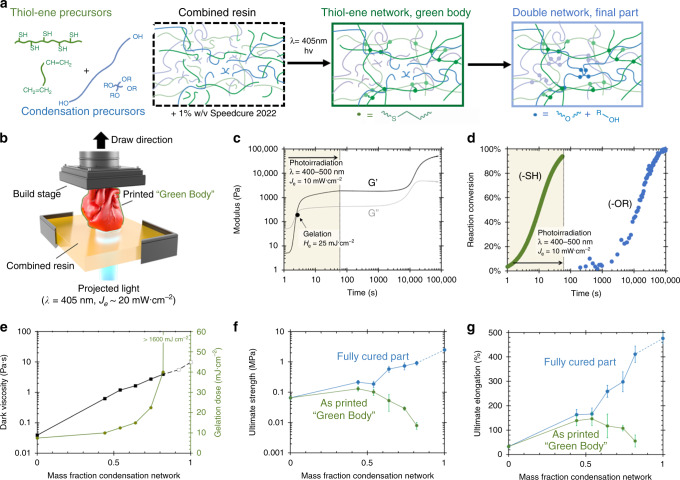
3D printing double network silicones (SilDNs). **a** The precursors to a photocured thiol-ene silicone (green species) and a condensation silicone (blue species) combine to create the 3D printing resin. When exposed to light, the thiol-ene network crosslinks to form a green body that traps in the condensation components that slowly crosslink at room temperature to form the second network. These reactions are orthogonal and yield two distinct networks that occupy the same volume. **b** A schematic overview of the stereolithography (SLA) printing process. **c**, **d** The 82%MM10T resin’s rheological behavior (**c**) and the chemical conversion of crosslinkable groups (green circles denote thiol groups; blue circles denote alkoxy groups) (**d**) shows the independent development of the two networks during the printing process. **e** The dark viscosity (black line) and gelation dose (green line) of SilDN resins as a function of the relative mass fraction of the two networks. Open symbols denote compositions that are unprintable (*N* = 3). **f**, **g** Ultimate strength (**f**) and elongation (**g**) in the as-printed green body (green line) and final part (blue line) as a function of mass fraction of the two networks. Error bars represent the SD in measured values (*N* > 7).

An alternative toughening strategy, particularly common in hydrogels, creates interpenetrating polymer networks (IPNs)^17^ or double networks (DNs)^18^ wherein two distinct percolated networks of polymers homogeneously occupy the same volume. The fracture of the brittle network dissipates energy, whereas the secondary network can remain intact and sustain loading. We propose a family of silicone DNs (SilDNs) composed of a weak, but 3D-printable silicone network that ensnares the precursors to a commercially available mechanically robust RTV silicone (Fig. 1a). This materials platform can simultaneously possess low elastic moduli (100 kPa < *E*_100%_ < 670 kPa) and a high elongation (d*L/L*_0_ ~ 400%), toughness (*U* > 1 MJ m^−3^), and strength (*σ* ~ 1 MPa) previously inaccessible in SLA elastomers. Printed objects from this family can also bond to other suitable substrates (thermoplastics, thermosets, ceramics, and metals) across seven orders of magnitude in Young’s modulus (10^4^ ⪅ *E* ⪅ 10^11^ Pa) regardless of the manufacturing process to form functional multi-material assemblies.

## Results

### Material design

Based on previously published work, we selected a thiol-ene silicone formulation that possessed excellent printability (low viscosity, rapid gelation, and high reaction conversion)^11^. In this click reaction, a carbon-based sulfhydryl (R-SH) group adds to an alkene (C = C) to form an alkyl sulfide [Eq. (1)].

1 $${\mathrm{R}} - {\mathrm{SH}} + {\mathrm{R}}^{\prime} - {\mathrm{CH}} = {\mathrm{CH}}_2\mathop{\longrightarrow}\limits^{{hv}}{\mathrm{R}} - {\mathrm{S}} - {\mathrm{CH}}_2 - {\mathrm{CH}}_2 - {\mathrm{R}}^{\prime}$$

Unlike other photoinitiated free-radical polymerization chemistries, thiol-ene reactions are thermodynamically favorable (*ΔH*_thiol-ene_ ~ 3 · *ΔH*_acrylate_) stepwise addition reactions that occur with rapid kinetics to high yields and do not rely on the propagation of a growing radical chain^19^. A bottom-up SLA printer with a transparent polymethylpentene (PMP) window (Fig. 1b) can successfully print thiol-ene-based poly(dimethylsiloxane) resins^11^.

The second polymer of the DN system must form its own distinct network, e.g., it must undergo a chemically orthogonal crosslinking reaction. Vinyl groups (−CH=CH2), such as those in platinum-cured RTVs, can participate both in hydrosilylation and free-radical photopolymerization. Thus, these vinyl-bearing species would be incorporated into the photopolymers to form a single polymer mesh. To avoid this unwanted interaction, we restricted the secondary network to condensation silicones, which do not participate in photochemistry, as previously demonstrated^20^. The Mold Max (MM) Series (Reynolds Advanced Materials) are commercially available silicones with industrially relevant strength, elongation, and toughness over a range of elastic moduli (250 kPa < *E* < 2 MPa). As written in Eq. (2), these materials possess hydroxyl (R-OH) groups that react with an ether (R-O-R) in the presence of a tin catalyst to release byproducts of hydrolysis, usually water, methanol, ethanol, or acetic acid (H-OR) over the course of hours.

2 $${\mathrm{R}} - {\mathrm{OH}} + {\mathrm{R}}^{\prime} - {\mathrm{OR}}\prime\prime \mathop{\longrightarrow}\limits^{{{\mathrm{Sn}}\,{\mathrm{atalyst}}}}{\mathrm{R}} - {\mathrm{O}} - {\mathrm{R}}\prime + {\mathrm{H}} - {\mathrm{OR}}^{\prime\prime}$$

Figure 1a depicts the reaction scheme of our system. These materials, when combined, form sequentially IPN^17^: a photocured thiol-ene silicone and a mechanically robust condensation-cured silicone.

### Photorheology

Photorheology characterized the printability of our resin system. We infer the liquid-to-solid transition from the gel dose or the photo-irradiation necessary for the storage modulus (G′) to exceed the loss modulus (G″), i.e., when the mechanical energy is stored elastically more than dissipated viscously^21^. As shown in Fig. 1c, the material (82%MM10T) rapidly gels as noted by the crossover in moduli at a modest photodose (*H*_e_ ~ 25 mJ cm^−2^) corresponding to only a few seconds of exposure that is similar in spectrum and intensity to common SLA printers (400 < *λ* < 500 nm, *J*_e_ ~ 10 mW cm^−2^). The moduli quickly begin to plateau with continued irradiation, suggesting that the polymerization of the thiol-ene network nears completion. After this first minute, we cease exposure and continue to track the rheology of the green body (i.e., the object immediately after photocure but prior to additional processing steps). As the condensation reactions proceed in the dark, both moduli experience an additional sharp rise and plateau hours later corresponding to the formation of the second network.

Figure 1d further highlights that the orthogonal chemical reactions and difference in relative reaction speeds allow for the sequential fabrication of the two networks. In situ tracking of functional groups via photo-differential scanning calorimetry (photo-DSC) and Fourier transform infrared spectroscopy (FTIR) enable measurement of the depletion of crosslinking groups. Consistent with the high yield expected in click chemistry, the initial photoirradiation (*H*_e_ ~ 600 mJ cm^−2^) consumes 94% of the thiol groups during the formation of the green body (Supplementary Fig. 1). Similarly, the observed conversion of –OR groups corresponds in time to the formation of the second set of plateaus in G′ and G″. These initial experiments and the glass transition measurements (Supplementary Fig 1c) confirm that the intersystem crosslinking is negligible, which preserves the individual performance of the two constituent systems.

In addition, the relative mass fraction of the two networks can be selected to tune the resin’s printability and the material’s mechanical performance. Both parent materials possess a siloxane backbone with the majority of side groups being methyl (>97 mol%), excepting for the small fraction of functional groups utilized for crosslinking (e.g., –ROH, –OR’ in condensation-cured silicones and –(CH_2_)_3_SH and –CH=CH_2_ in the thiol-ene resin). The presence of large dimethylsiloxane segments in both networks imparts the miscibility required for producing homogenously interpenetrating networks. As shown in Fig. 1e, the lack of phase separation also enables reducing the apparent viscosity of the more viscous condensation-cured precursor (*η*_0,Mold Max 10T_ = 9.8 Pa s) with dilution by the less viscous thiol-ene resin (*η*_0,thiol-ene_ = 0.04 Pa s). Even at relatively high mass fractions (~85 wt%) of the condensation network, the combined viscosity remains in the regime suitable for stereolithographic printing (*η*_0,combined_ < 5 Pa s). Regardless of viscosity, resins where the condensation network exceeds this loading (85 wt%) are unprintable; the thiol-ene network cannot span the entire volume and achieve the percolation necessary for gelation at reasonable photo doses (>1600 mJ cm^−2^).

As parts need to survive the stresses of the printing process (Supplementary Video 1), we note the mechanical performance of both the final material and the photocured green body. As photo-exposure forms the thiol-ene network, an increasing mass fraction of condensation network decreases the strength of the green body. However, the increased loading of the condensation network eventually provides for a much stronger final part. As shown in Fig. 1f, we observed this effect being most pronounced at 82 wt% of condensation network, where green body undergoes a greater than two orders of magnitude increase in ultimate strength as the second network forms in the final part (*σ*_green body_ = 0.008  MPa, *σ*_final part_ = 0.92 MPa).

Previously reported two-stage resins can similarly undergo further reaction from the printed green body to yield an increased strength, but these reported materials form a single polymer^22^. Consequently, the second stage of polymerization increases the crosslink density of the network, which stiffens and embrittles the material. As evidenced in Fig. 1g, by forming two distinct networks, we maintain the low effective volumetric crosslink density of each individual network and preserve a large molecular weight between these crosslinks. At high mass fractions of condensation material, these molecular segments can uncoil to increase the ultimate elongation by 12-fold over the pure thiol-ene material ([d*L/L*_0_]_thiol-ene_ = 0.33, [d*L/L*_0_]_82MM10T_ = 4.11). Although such a high condensation loading yields superior mechanical properties, other intermediate blends may still be desirable to print. Particularly, the combination of lower viscosity, faster gelation, and a stronger green body makes compositions with increasing thiol-ene content easier to rapidly print. This lower starting viscosity also corresponds to a greater printing pot life from a single batch of mixed resin (Supplementary Fig. 2). Furthermore, while meeting the process requirements of SLA, these SilDNs are also compatible with less restrictive additive manufacturing techniques like UV-curing injection molding.

### Mechanical properties

**Fig. 2:**
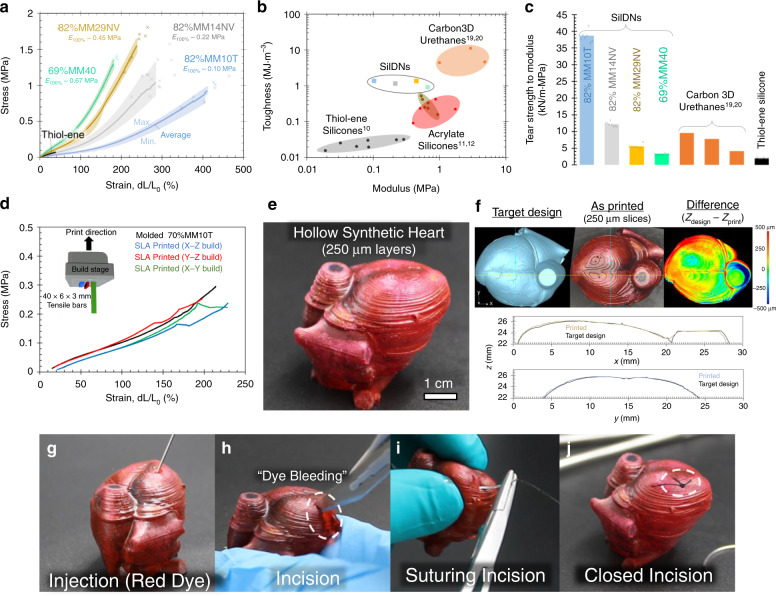
Mechanical properties of SilDNs. **a** Combined tensile test data for the toughest printable material from each base condensation network (MM10T in light blue, MM14NV in gray, MM29NV in gold, and MM40 in green). Lines depict the average curve (*N* > 7), shaded regions correspond to max and min stresses at each strain value, and “x” denotes actual failure points for each material. **b** Toughness vs. modulus for SLA-compatible silicones and commercial SLA polyurethanes (*N* > 7). Material families are grouped by the shaded regions. **c** Normalized tear strength of double network silicones and commercial SLA polyurethanes with overlaid data points (*N* = 7) for custom each material. **d** Average stress–strain curves for 70%MM10T as fabricated by different SLA print orientations (*X*–*Z* build direction is the blue curve, *Y*–*Z* is red, *X*–*Y* is green) and molding (black curve) showing little anisotropy (*N* > 3). **e** A 3D printed (64%MM10T) surgical simulator that replicates the organ morphology and elastic modulus of cardiac tissue. **f** Horizontal (yellow) and vertical (blue) topologies of the printed heart closely replicate that of the target design (black lines) as measured by laser confocal microscopy. **g**–**j** This hollow device enables simulation of numerous surgical skills. The high tear strength of the base material enables closing the incision with sutures.

In addition to changing the relative mass fractions of the two networks, altering the base material of the condensation silicone enables another level of control over the mechanical performance of the SilDN material. There are numerous commercially available tin-cured silicones, including alternatives within the MM family, which possess distinct optical, rheological, and mechanical performance. We explored four candidate materials (MM10T, MM14NV, MM29NV, and MM40), where the numerical distinction corresponds to the manufacturer’s specified shore A hardness in the cured material (T denotes transparency and NV denotes a low-viscosity resin). Although the choice of MM silicone influences the photorheology of the blended resin (Supplementary Fig. S3), the effect is slight—a similar regime of printable blends emerged. If the weight fraction of the thiol-ene network exceeds a critical threshold (~15 wt%), the viscosity and photoirradiation dose for gelation remain low enough for conventional SLA printing. As shown in Fig. 2a, the tin-cured component dominates the mechanical performance. For all of these SilDNs, the toughness and strength increased by orders of magnitude (0.92 MJ m^−3^ < *U* < 1.37 MJ m^−3^, 0.92 MPa < *σ* < 1.54 MPa) from the pure the thiol-ene material (*Γ*_thiol-ene_ ~ 0.02 MJ m^−3^, *σ*_thiol-ene_ ~ 0.07 MPa). For simplicity, we use the following naming convention: “XX%MMYY,” where “XX%” is the mass fraction of the condensation network and “MMYY” is the grade of MM silicone. Supplementary Fig. 4 contains full mechanical data for all blends as well as the extractable gel fractions for select compositions of SilDNs.

Figure 2b compares our SilDNs to other available SLA silicone^11–13^ and polyurethane elastomers^23,24^. The other SLA silicone families (grouped by shaded areas) offer the ability to tune mechanical performance by increasing or decreasing the crosslink densities. This conventional strategy introduces a trade-off between softness and toughness—soft materials are generally delicate and vice versa. However, our chemistry consistently maintains a toughness an order of magnitude greater (*U* ~ 1 MJ m^−3^) than similarly soft SLA materials (100 kPa < *E* < 670 kPa). Further, as in other DN systems^18^, Fig. 2c shows the normalized tear strengths of SilDN are large and can exceed that of even commercial polyurethane SLA elastomers^23,24^. The combination of low moduli, high toughness, and high tear resistance is desirable when printing soft robotic and biomedical devices^2^. Unlike other materials where the 3D printing process can impart anisotropy or alter performance, SilDNs can possess similar properties regardless of print orientation or layer height (Fig. 2d). These findings suggest that the condensation network crosslinks across printed layers.

### 3D printing SilDNs for surgical training and biomedicine

Medical and surgical simulators are valuable tools to train practitioners, plan surgery, and inform patients of procedures. Extra-clinical surgical simulation improves performance and patient outcomes while reducing expensive operating time and the rate of intraoperative errors^25^. Silicone materials can simulate tissue mechanics^26^ and commercial silicone-based suture pads exist to rehearse clinical skills. Unfortunately, such devices fail to fully replicate actual surgical conditions; human tissues are often arranged in intricate architectures that deform considerably when manipulated. These adverse conditions lead novice surgeons to poorly place the needle, misalign stitches, and improperly balance tension in the thread^27^. To demonstrate our chemistry’s ability to affect these applications, we printed a hollow synthetic heart model using 64%MM10T similar in scale to an infant’s heart (Fig. 2e). The resolution of the print process is such that final object closely resembles our target design based on 250 μm layers (Fig. 2f, Supplementary Fig. 6, and see Supplementary Information for more details on resolution). Although unable to fully replicate the complex mechanical performance of natural tissue (anisotropy, fiber orientation, etc.)^28^, this composition possesses an elastic modulus similar to cardiac tissue (*E*_100%_ ~ 100 kPa). Like previously demonstrated 3D printed organ phantoms^29,30^, these devices allow care-givers to practice numerous skills such as injection, incision, and suturing (Fig. 2g-j). Particularly enabling is the material’s high tear strength, which allows the thin walls to survive the local stresses imparted, while sewing and securing the suture knot (Supplementary Video 2). In this demonstration, we estimate a tear resistance exceeding 2.5 kN m^−1^ based on an estimated max reactive force of ~5 N existing within the 2 mm heart walls^27^.

### Bonding 3D printed assemblies

For many multi-material devices containing elastomers, no single continuous additive manufacturing process can fabricate all the individual components of most commercial products. A primary cause of this issue is that strategies compatible with thermosetting elastomers (molding, direct ink writing, ink jetting, and SLA) are incongruous with conventional methods for fabricating thermoplastics, metals, and ceramics (rolling, molding, fused deposition modeling). Moreover, the current economics of large-scale manufacturing of elastomers still favor conventional strategies (e.g., injection molding) over 3D printing except for small, intricate architectures (i.e., unmoldable geometries). In this regard, silicones are particularly restricted; the high extensibility of the material and the chemical inertness of the siloxane backbone makes intra- and inter-layer adhesion of prefabricated components weak.

**Fig. 3:**
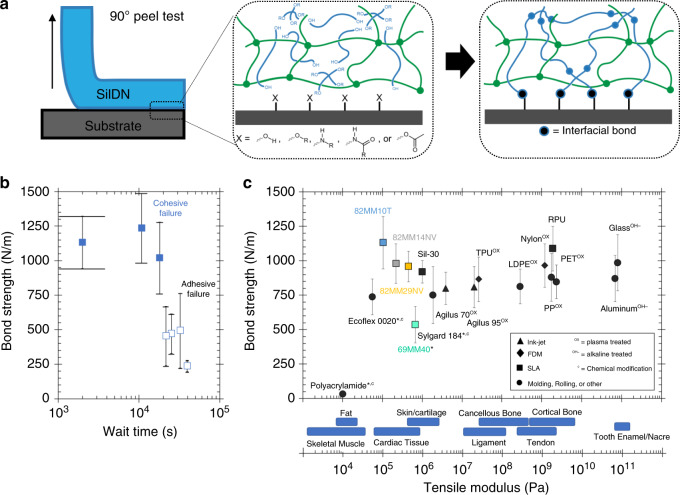
Post-print bonding of SilDNs. **a** Overview of 90° Pee Test and proposed schematic of bonding process. Numerous condensable groups (*X* = –OH, –OR, –NHR, –NHR[C=O]R, –O[C=O]CH_3_) can participate in the proposed interfacial bonding reaction. **b** Bond strength vs. wait time for 82%MM10T bonded to itself. Open symbols denote adhesive failure, closed symbols denote cohesive failure. The height of the error bars corresponds to the SD and the width of error bars denotes uncertainty in wait time (*N* > 7). **c** Bond strength for 82%MM10T to numerous substrates (*N* > 7 for each material combination). Materials* whose ultimate strength is below that of the SilDN display a lower bond strength as a result of cohesive failure through the substrate itself. Superscripts denote any modifications to the substrate that improved bondability. Triangle symbols represent ink-jetted samples, diamonds represent FDM printed samples, squares represent SLA printed samples, and circles represent conventionally fabricated (molding, rolling, or other) samples.

The SilDN chemistry presented here improves this complex bonding issue. As outlined in Fig. 3a, the slower kinetics of the latent condensation-cured network allows us to bond SilDN green bodies across interfaces to other substrates with condensable groups (–OR, –OH, etc.). For 82%MM10T, the density of unreacted groups (six hours post fabrication) remains high enough for cohesive bonding (Fig. 3b). In addition, such bondable groups either exist innately or can be induced [e.g., chemical treatment (^c^), plasma oxidation (^ox^), alkaline treatment (^OH−^), etc.^31–33^] onto diverse classes of materials. Taking advantage of this chemistry, we bonded the 3D printed SilDNs onto ceramics (glass), metals (aluminum), common thermoplastics (polypropylene [PP], polyethylene [PE], poly(ethylene terephthalate) [PET], thermoplastic polyurethane [TPU]), hydrogels (polyacrylamide), platinum-cured RTV silicones (Ecoflex 0020, Sylgard 184), and other thermosetting polymers (rigid polyurethane [RPU], nylon, urethane acrylate [Agilus 70 and Agilus 95], silicone urethane [Sil-30], and other SilDNs). Figure 3c shows that all bonded samples in the peel test failed cohesively, or through the bulk of the weaker substrate, rather than adhesively, or at the interface (see Supplementary Video 3 for more details). For polyacrylamide*, Ecoflex 0020*, and 69%MM40*, the lower reported bond strength is still cohesive; we attribute this marked decrease in bond strength to the inferior ultimate strength of the substrate (Supplementary Video 3). For the remaining materials, the strength of the SilDN material limited the measured bond strength (*Γ* ⪆ 800 N m^−1^). The bond strength did not decrease when aged at ambient conditions over 2 months (Supplementary Fig 5).

**Fig. 4:**
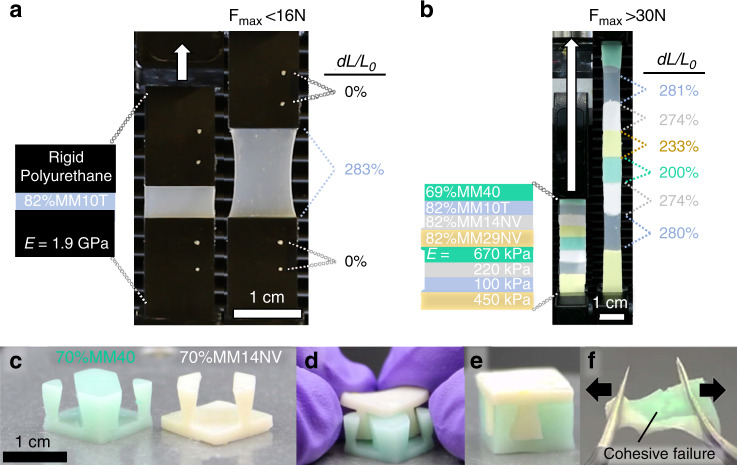
Multi-material assembly of SilDNs for programmed mechanical gradients. **a** A photocured 82%MM10T (*E*_100%_*=* 100 kPa) block is attached to rigid polyurethane segments to form a tensile coupon (3 mm thick) with a steep modulus gradient. When loaded, the strain is localized entirely to the soft SilDN section (calculated visually, white dots added for tracking). The concentration of stress at the interface causes failure prior to reaching 16 N of applied force. **b** SilDNs are combined post photocure to form a tensile coupon with a shallow modulus gradient. The relative strain of each segment is inversely proportional to the stiffness of the SilDN component. The smooth gradient allows this coupon to sustain a load in excess of 30 N. **c** A pair of objects containing complementary tapered features (dovetails) printed from different SilDN materials. **d** Aligning the compliant green bodies to form a mechanical interlock. **e** The assembly after fully curing the SilDN components shows **f**, cohesive failure during destructive testing.

As denoted by the different symbols in Fig. 3c, bonding is maintained regardless of substrate manufacturing method. Combining manufacturing processes is a pragmatic benefit when assembling devices, as certain subcomponents are more readily fabricated with specific techniques^2^. This bonding process is also insensitive to the substrate’s Young’s modulus, allowing us to create tunable stiffness gradients in post-print assemblies that vary over seven orders of magnitude. Interestingly, the mechanical gradients employed in animal physiology span an almost equivalent range from tooth enamel (10^11^ Pa) to fat (~10^4^ Pa), as shown by the second horizontal axis in Fig. 3c^34–36^. Multi-material assembly of SilDNs enable programmed mechanical gradients that can control the mechanical response of the resulting object and mitigate failure under load. In Fig. 4a and Supplementary Video 4, a soft SilDNs structures permits extreme strain localization to protects brittle components (RPU) at modest loads (*F* < 16 N). Conversely, a shallow gradient (Fig. 4b and Supplementary Video 4) minimizes stress concentrations and delays failure (*F* > 30 N)^37^. In addition, Fig. 4c–f highlights the unique ability of SLA to fabricate small (~10 mm), compliant structures with overhanging features. We use two different SilDN materials to create complementary mortises and tenons, a common design for increasing the tensile strength of joined objects. In this example, the structures mechanically interlock upon assembly and the SilDN green bodies further bond post print. Even when the dovetail joint is intentionally destroyed, the interface maintains cohesive failure (Fig. 4f and Supplementary Video 5).

### Integration of SilDN actuators into wearables and robotics

**Fig. 5:**
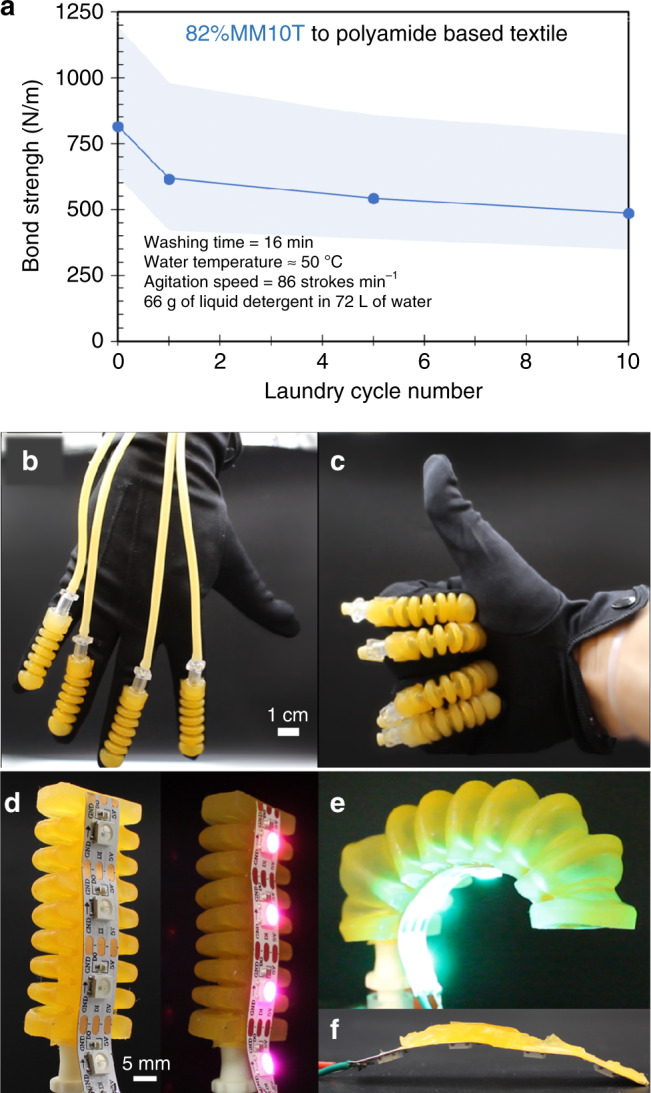
Integration of SilDNs for wearable and soft robotic applications. **a** Bond strength of SilDN-polyamide-based textile assembly as a function of laundry cycle. All samples failed cohesively with the shaded region denoting the standard deviation in bond strength (*N* > 7). **b** Four 3D printed fluidic actuators bound to a Nylon-based garment to create a low encumberance orthotic glove. **c** The direct integration of such soft actuators maintains dexterity of the user. **d** A 3D printed bellows actuator containing a flexible PCB with LEDs as the strain limiting layer. The low temperature bonding process preserves the functionality of LEDs. **e** The interfacial bond between the components survives large actuation amplitudes. **f** Intentional destruction of the device shows cohesive failure of the SilDNs and adhesive failure at the interface.

Functional, wearable soft actuators and sensors can recognize body poses^38^, assist with manipulation^39^ or ambulation^40^, and generate haptic information to a user^41,42^. As unconstrained soft matter deforms continuously, such devices need to be properly grounded to measure or generate appreciable, localized forces.^41^ Thus, a strong elastomer-textile bond can help overcome key challenges to wearable devices that impart perceptual forces onto the human. Tear resistant enough to bear a stitch for mechanical integration (see Fig. 2i, j above), SilDNs can also bond to common textiles such as Nilo^TM^, a stretchable fabric based on 86% polyamide and 14% elastane (Fig. 5a). Such assemblies again exhibit a strong cohesive bond (*Γ* ⪆ 800 N m^−1^). When subjected to relatively severe home laundering conditions according to AATC 124 (see “Methods” for experimental details), the bond strength does drop to *Γ* ~ 550 N m^−1^. Despite the reduction in bond strength, the failure mode is still cohesive in nature (Supplementary Video 3). This result suggests compatibility with the requirements for consumer-grade, reusable smartwear. Figure 5b shows such a device: an orthotic glove with four printed pneumatic actuators located on the dorsal side of the interphalangeal joints cohesively bonded to a polyamide based glove. Here, the soft silicone actuators do not significantly encumber the user and allows for dexterous manipulation (Fig. 5c and Supplementary Video 6), while also preserving the benefits of the substrate fabric (e.g., appearance, breathability, and feel). Such bonded structures continue to survive hundreds donning and doffing cycles (Supplementary Video 7) over 10 months after assembly.

Latent reactive bonding is likely possible in other two-stage curing materials; however, the condensation reaction in SilDNs advantageously proceeds at room temperature to permit processing thermally sensitive components. Figure 5d highlights this capability by affixing a 3D printed bellows actuator to a commercially available flexible printed circuit board (PCB, Kapton^TM^) containing temperature sensitive light-emitting diodes (LEDs, *T*_max_ ~ 80 °C). This electronic component not only survives the bonding process, as evinced by the lit LEDs (Fig. 5d), but functions as the strain limiting layer that directs the bending motion of the actuator (Fig. 5e). As shown in Supplementary Video 8, the bond between the SilDNs and PCB survives numerous actuation cycles to large strain states without delamination. Even during over pressurization, the membrane ruptures prior to failure of the interfacial bond. A final, manual, attempt to separate the bonded surfaces shows cohesive failure of the SilDN layer rather than adhesive failure at the SilDN-PCB connection (Supplementary Video 8 and Fig. 5f). As custom electrical circuits are easily produced commercially on PCBs, this simple device implies the broader potential for SilDNs to integrate the rigid elements required for power, sensor, and computing systems within softer robotic bodies.

## Discussion

We have created an SLA printable SilDN chemistry that possesses superior mechanical properties (d*L/L*_0_ < 400%, *U~* 1.4 MJ m^−3^, *σ* ~ 1 MPa) than other printable elastomers with a similar elastic modulus (*E*_100%_ < 700 kPa). The framework of this approach allows for independent control of the transient (processing) and steady state properties of the material: a thiol-ene network, which provides shape fixity during printing, and a condensation-based network, which forms later to dictate the mechanical behavior. The composition and relative ratio of these two networks determines both the printability (photopolymerization rate, viscosity, green strength) and the mechanical properties of the printed part allowing for tunable performance. The latent reaction offers an opportunity to form cohesive bonds between SilDNs and disparate substrates spanning the seven orders of magnitude in modulus—comparable to what is found commonly in biology. We further use this ability to robustly attach soft actuators onto garments for wearables, or with temperature sensitive electrical components for integrated robotics.

By introducing the SilDN framework, we note opportunities to improve process speed and final material properties. While the photopolymerization kinetics of the thiol-ene network enable rapid 3D printing, the tin-catalyzed condensation network forms over a period of hours to prolong total manufacturing time. Increasing the polyermization rate of this second condensation network would decrease both the pot life of the resin and the time-window for assembly and bonding. Looking forward, we suggest photo-latent catalysts^43^ to control the condensation reaction for either discrete bonding steps or simultaneous DN formation during printing. Further, the photocurable network can be made stiffer and stronger than the soft and highly extensible condensation network to increase toughness and green body strength. This approach would mirror previously reported hydrogel DNs that combine mechanically disparate polymers, and could lead to SilDNs that greatly outperform both constituents^18^. Preliminary investigations of known stiffer SLA silicone photochemistries^11,12,14^ reveal that such blended resins lack the proper photorheological and mixing properties for SilDN printing, though alternative systems may meet this target. SilDNs can also be produced from other condensation networks including those with advanced functionality (e.g., self-healing behavior) to enable a device performance^44^.

## Methods

### Preparation of silicone resins

To create a SilDN resin, we add the two-part condensation-cured silicone (MM10T, MM14NV, MM29NV, or MM40) as per the manufacturer’s (Reynold’s Advanced Materials) recommendation. Then, immediately after the addition of catalyst, we combine the condensation resins with the appropriate mass fraction of previously prepared thiol-ene resin and liquid photinitiator (1 mL Speedcure 2022, Lambson, Inc., per 100 g of thiol-ene resin). As reported^1^, the composition of the thiol-ene resin is 61.7% by wt vinyl terminated poly(dimethylsiloxane) (Mw ~ 6000 kDa, Gelest, Inc.) and 38.3% by wt [4–6% (mercaptopropyl)methylsiloxane]-dimethylsiloxane copolymer (Gelest, Inc), which corresponds to 1 : 1 vinyl to thiol stoichiometry. A centrifugal mixer (Speedmixer DAC 600.2 VAC-LR FlakTek) blends all materials at 1500 r.p.m. for 35 s followed by 2000 r.p.m. for 55 s.

### Photorheology

A rheometer (DHR3, TA instruments) with a photo-curing accessory (TA instruments) measured the curing behavior of the silicone resin. An oscillatory shear experiment rotated parallel plates (diameter = 20 mm) at constant frequency (*ϖ* = 2 Hz) and amplitude (*γ* = 1%) tracked the evolution of viscosity and the complex moduli. The bottom plate of the photo-curing accessory is a transparent acrylic plate that is coupled to a lightsource (Omnicure Series 1500, Lumen dynamics) and filter (*λ* = 400–500 nm). The experiment is begun in the dark, followed by a period of photo-irradiation. The power density on the sample during illumination was set to be *J*_e_ = 10 mW cm^−2^ as measured by a Silver Line UV Radiometer (230–410 nm). To minimize the effects of absorption, the gap thickness was set to the minimum value, *t* = 250 µm, which yielded consistent results. Data for each composition were collected in triplicate with the average reported. Dark viscosity was determined by the average viscosity 10 s prior to illumination.

### Fourier transform infrared spectroscopy

We infer reaction extent for the condensation reaction by using infrared spectroscopy by first placing a small aliquot of uncured 18%MM10T material on top of an IR-transparent KBr cell. A Bruker Lumos FTIR Microscope measures the initial material spectrum in transmission (64 scans, 600–4000 cm^−1^) Then, photo-irradiation (Omnicure S2000, 1000 mJ cm^−2^, *λ* = 400–500 nm) cures the material into a green body. Repeated measurements are collected at intervals for the next 30 h. The decrease in the 2855–2825 cm^−1^ peak corresponds to the consumption of alkoxy groups (–OCH_3_) as quantified by a sigmoidal integration and normalized by a standard peak from 2440 to 2540 cm^−1^. We obtain the condensation network’s reaction conversion by taking the peak area at each time and dividing by the initial alkoxy peak height prior to photo-irradiation.

### Photo-differential scanning calorimetry

The low density of thiol and vinyl groups in the 18%MM10T resin prevents tracking the conversion of the thiol-ene resin by FTIR. Instead, photo-DSC enables measurement of the extent of reaction by tracing the photopolymerization exotherm. We transfer approximately 30 mg of 18%MM10T resin into an uncapped aluminum pan. Two waveguides coupled to the instrument transmit equal photo doses (*J*_e_ ~ 10 mW cm^−2^, *λ* = 400–500 nm) to the sample pan and an empty reference pan, while the DSC measures heat flow. Photo exposure occurs in a pair of 1 min pulses, with 1 min of dark in between. Additional pulses do not result in any additional curing as noted by the exotherm peak size being consistent. To remove the contributions to heat by the photo-irradiation, the exotherm from the last pulse is subtracted from the first. We attribute this normalized heat flow solely to the heat of reaction for the thiol-ene photopolymerization (*ΔH*_rxn_ ~ 60 kJ mol^−1^ SH). Calculating a running integral of the exotherm on a thiol content basis (3.94 × 10^−5^ mol SH per g resin) and dividing by the heat of reaction^1^ yields the thiol-ene network’s reaction conversion as a function of exposure time.

### Tensile tests

We slowly pour the mixed resin into an aluminum sheet mold (3 mm × 130 mm × 200 mm). A vacuum of 29+ mm Hg is pulled for 1 min For optical transparency, 4 clamps tighten an acrylic sheet on top of the resin. UV light (Uvitron PortraRay 400 W UV Lamp) cures the resin sheet for 5 min (measured irradiant flux of *J*_e_ ~ 26.6 mW cm^−2^, *λ* = 230–410 nm). From here the material is removed from the mold and tested as a green body state or fully cured at room temperature for 16 ± 4 h with an additional 2 h at 65 °C. An Instron ASTM die C cutter (gauge length 33 mm, width 6 mm) punches tensile coupons from the sheet of material. We then draw two dots along the gauge length, ~25 mm apart for optical tracking with a video extensometer attachment. Then mount the dog bones in the Instron Universal Testing System (model 5943) series held by pneumatic clamps at a pressure, *ΔP* = 1 psi. During the test, we pulled the samples at a rate of 75 mm min^−1^. We calculate the Young’s Modulus (*E*_100%_) over the 5–100% strain regime. Tear test sheets are manufactured using the same process and then cut using ASTM Die B. The tear pull-rate is 500 mm min^−1^ per ASTM D624. We conduct each measurement at least seven times per material.

### Laser confocal microscopy

We used a Keyence VR-3200 G2 series microscope to directly measure the dimensions of printed parts. In addition to wide area 3D measurements, the instrument software can also compare a measured 3D profile to a reference CAD model. We directly uploaded the print file and used the auto-align feature to generate comparisons between the as measured part and the original CAD file.

### 90° Peel tests

For SilDN bonding, we pour the appropriate mixed resin into a transparent acrylic Substrate A mold (base coupon, 50 × 35 × 3mm) and Substrate B mold (peel coupon, 100 × 25 × 3 mm). The stiffer material is Substrate A, the softer material is Substrate B. We expose both molds to UV light (Uvitron PortaRay 400W UV Lamp) at an irradiant flux of *J*_e_ ~ 26.6 mW cm^−2^ as measured with a Silverline Radiometer (230–410nm), for at least 5 min to cure the green body network. An alignment mold places Substrate A and B into contact and the condensation network cures for over 12 h at room temperature followed by a 65 °C cure for 2 h. For other materials, we directly 3D print (Sil-30, RPU, TPU, and Nylon), cast (polyacrylamide, Sylgard 184, Ecoflex 0020), or machine (aluminum, glass, PP, low-density PE (LDPE), PET) the samples in the Substrate A geometry.

The innate condensable groups exist on Carbon Sil-30 and RPU materials require an elevated post-process thermal treatment to react (120 °C). To balance this treatment with our preferred method for curing the SilDNs, these materials remain in contact for 15 h at room temperature prior to heating per the Carbon material’s recommendation. Qualitatively, Carbon EPU also bonds cohesively to SilDNs, but the EPU material needs a high temperature cure within 8 h. Such a rapid heat treatment weakens the SilDN, but still permits cohesive failure of this weaker substrate.

Similar to previous methods^31^, chemical modification of acrylamide and platinum cured silicones impart condensable groups by mixing in small amounts of triethoxyvinylsilane (TCI America, 0.1 % by weight for Sylgard 184, 0.3% by weight for Ecoflex 0020) or 3-trimethoxysilyl propyl acrylate (TCI America, 0.1% by weight polyacrylamide) prior to cure. For solid materials without a high density of innate condensable groups, surface treatment permits bonding. Oxidative plasma treatment (1 min at 60W) of Nylon 12 (Stratasys, 0.010” resolution), Agilus 70 (Stratasy, glossy surface finish, 30 μm layer height), Agilus 95 (Stratasys, glossy surface finish, 30 μm layer height), TPU 95A (Ultimaker, 0.2 mm resolution), PP (McMaster Carr, 8742K235), LDPE (McMaster Carr, 8657K214), PET (Small Parts, PES-23900-F-01), Nylon-based glove (Amazon, ASIN: B01IBVCSC2), Polyamide fabric (NILO, Jersey Lomellina) samples followed by coating with poly-vinyl methoxy siloxane (Gelest, Inc.) and a 20 min stabilization at 80 °C yields surface condensable groups. For aluminum 6061-T6 (McMaster Carr, 9246K13) and borosilicate glass (McMaster Carr, 8476K21), we employ a previously established protocol to remove contaminants from the native oxide surface.^32^ Briefly, a minute long soak in a base bath (NaOH, pH ~ 10) followed by a Deionized H_2_O rinse and then a dip in a dilute 1,2, bistriethoxysilylethane solution (Gelest, Inc.) results in exposed ethoxy groups for condensation. After these modifications or treatments, samples are ready for bonding to the DN silicones as outlined above.

After bonding, we peel samples according to ASTM D3135. Briefly, a custom track and carrier hold peel test coupons and maintain the peel angle at 90°. An Instron Universal Testing System (model 5943) peels substrate B off A at 300 mm min^−1^, while recording Force for at least seven bonded samples. We normalize the maximum recorded force value by the width of the bond line (25 mm) to obtain the bond strength.

### Multi-material assembly

After printing objects, we cleaned green bodies with a quick (<1 min) isopropyl alcohol bath. We then placed objects on to substrates that were appropriately modified to enable bonding (see above) via the protocols above, if necessary. It is best if intimate contact is maintained during the room-temperature bonding step. We found that when joining thin walled fluidic actuators on to garments, the irregular shapes makes it difficult for this contact to maintained. Applying a small amount of uncured resin to problematic edges and spot curing with a UV wand (Omnicure S2000) can help keep soft, delicate objects in contact, while the condensation network cures. We note that this step is not always necessary and the bond that forms between the two bodies extends well beyond this temporary adhesive we apply.

### Laundering cycles

We based our laundering cycles from the AATC TM 124 standard for measuring the durability of fabrics subjected to home laundering. Our protocol utilized a hot water setting (water temp 49 ± 3 °C) with 66 ± 1 g of Tide^TM^ Free and Clear Phosphate Free Laundering Detergent in 72 ± 4 L of water. Each washing cycle consisted of 16 ± 1 min of agitation at 86 ± 2 strokes per minute followed by a spin cycle of 5 ± 1 min at 660 ± 15 r.p.m. Each load consisted of bonded assemblies and laundering ballast (Type III, 50% cotton/50%polyester) totaling 4.0 ± 0.2 lbs of total weight. Samples were then air-dried and subjected to 90° peel tests as outlined above.

*3D Printing*: We print all objects on a modified bottom-up commercial desktop SLA printer (Ember by Autodesk) using a blue-light LED projector (*λ* = 405 nm, *J*_e_ = 20 mW cm^−2^) modified to use a PMP window. We found that inclusion of a wiper blade assembly that sweeps across the build area between layers improved print reliability. It is possible to improve the *z*-axis resolution by incorporating dye species (e.g., adding 10 mg Sudan I in toluene [10% by wt] per gram of resin). For verisimilitude, we paint the surgical simulator with SilcPigs pigments post-printing.

## Supplementary information

Supplementary Information

Peer Review File

Description of Additional Supplementary Files

Supplementary Movie 1

Supplementary Movie 2

Supplementary Movie 3

Supplementary Movie 4

Supplementary Movie 5

Supplementary Movie 6

Supplementary Movie 7

Supplementary Movie 8

## Data Availability

The authors declare that the data supporting the findings of this study are available within the paper and its Supplementary Information files or from the corresponding authors upon reasonable request.
