# Supplementary material for: 3D printable tough silicone double networks
Source: Nat Commun. 2020 Aug 10;11:4000. doi: 10.1038/s41467-020-17816-y (PMC7417997; doi:10.1038/s41467-020-17816-y)
Supplement: Supplementary file 1 — Supplementary Information [file 41467_2020_17816_MOESM1_ESM.pdf]

Supplementary Information for 3D Printable Tough Silicone Double Networks

Thomas J. Wallin[1*], Leif-Erik Simonsen[1], Wenyang Pan[2], Kaiyang Wang,[2] Emmanuel Giannelis,[2] Robert F. Shepherd[3], Yiğit Mengüç[1*]

1. Facebook Reality Labs; Redmond, WA USA 98052
2. Department of Materials Science & Engineering, Cornell University; Ithaca, NY USA 14850
3. Sibley School of Mechanical & Aerospace Engineering, Cornell University; Ithaca, NY USA 14850

Correspondence and requests for materials should be addressed to:

ThomasWallin@fb.com

softrobot@fb.com

Supplemental Information

Glass Transition Temperatures: We prepared and cured thiol-ene, MM10T and 50%MM10T materials. Briefly, precursor resins were cast into a 3 mm mold and subjected to H_e~8000 mW·cm^{-2} of photoexposure (λ = 230 - 410nm) followed by 16 h of RT cure and an additional 2 h of post-cure at 65°C. To extract any unreacted components or plasticizers (see Gel Fraction, below), we soaked all samples in toluene for 24 hours and dried samples in a vacuum oven at 80°C. From these cured specimens, we removed and massed small amounts of material (~10 to 20 mg) and placed these samples within sealed aluminum pans. Using a DSC (Q2000 series, TA instruments) with attached liquid nitrogen cooling system (LNCS, TA instruments), we varied the temperature from -180°C to 50°C at 10°C min^{-1} under a flow of nitrogen gas (50 mL min^{-1}). During this temperature ramp, we tracked heat flow into the sample pan and compared it to an empty, sealed reference. Supplementary Figure 1c shows the measured resulting exotherms from -170 to -110°C as high temperature features were not indicative of glass transitions. We utilized the Universal Analysis Software package (TA instruments) to measure any glass transitions (measured at midpoint). Both the temperature range selected and the transition's onset and offset (calculated) are recorded directly on Supplementary Figure 1c.

Unfortunately, for the pure MM10T, we observed no prominent glass transitions over the experimental regime. However, both the thiol-ene and 50%MM10T show sharp ($T_{g,onset} - T_{g,offset}$ = 2.8 °C), glass transitions at similar temperatures ($T_{g,thiol\text{-}ene}$= -124.8 °C, $T_{g,50\%MM10T}$ = -125.8 °C). Not only is this temperature consistent with silicones, but it is interesting to note that magnitude of the transition scales with the weight fraction of the thiol-ene component. This result suggests that the thiol-ene network possess a similar effective molecular weight and free volume in both the pure and combined systems, consistent with orthogonal double networks.

Gel Fraction Measurements: We first fabricated samples of the pure thiol-ene material, pure Mold Max materials, and varying composition of SilDNs as outlined above (i.e, H_e~8000 mW·cm^{-2}, λ = 230 - 410nm of photoexposure followed by 16 h of RT cure and 2 h of cure at 65°C). We then used a biopsy punch (~5mm diameter) to remove three separate cylindrical samples from each material. We record the original weight of all the disks (W_0) and then soaked in a vial containing acetone (20 mL) with moderate shaking for 24 hours at room temperature. After removing the unreacted precursors and additives, we thoroughly dry samples for 24 hours in an evacuated atmosphere at 80°C. The Gel Fraction (F_{gel}) is calculated by comparing the final weight (W_n) to the original weight ($F_{gel}= W_n/W_0$).

The results in Supplementary Figure 4e show a gel fraction of ~92% for the pure thiol-ene material, consistent with estimates for thiol conversion by photo-DSC (Figure 1d). Similar to many other commercial elastomers, the Mold Max series does incorporate plasticizer in the pure compositions, and, unsurprisingly, the amount of extractable plasticizer is inversely proportional to the stiffness and extensibility of these materials (i.e, $F_{gel,MM10T} < F_{gel,MM14NV} < F_{gel,MM29NV} < F_{gel,MM40}$). This data also further supports our claims of orthogonal reactions. It is interesting to note that the amount of extractable material in an individual composition appears to be a linear combination of the pure components. Thus, the thiol-ene reaction is not significantly altering the conversion extent of the condensation reaction and vice-versa.

Printed Resolution: There are numerous variables which impact printed resolution including considerations from the hardware (e.g. pixel size of the light source, exposure dosage, z-step

height, etc.) and the material resin (e.g. absorptivity, green-body strength, gelation dosage, shrinkage during polymerization, etc.).

Hardware Limitations to Green-Body Resolution: We used an Autodesk Ember to print our objects. The 64 by 40 print area is illuminated by a LED projector containing 1280 by 800 pixels. This corresponds to a pixel size of roughly 50 µm x 50 µm. Additionally the z-stepper motor on the Ember set to control the height of the build stage down to 10 micron increments. In practice we find that the obtainable resolution not restricted by hardware limitations.

Material Limitations to Green-Body Resolution: For SLA printing, the resolution in the z-direction (defined as parallel to the draw direction or incident light) depends strongly on the absorptivity of the resin, the gelation dosage, and photodosage provided for each build layer. As light penetrates the resin, it is absorbed according to Beer-Lambert law. Eventually the irradiative energy (H_e) of light falls below a critical value needed to initiate enough polymerization for gelation.[4] This depth is called the cure depth, or C_d. For our materials, we measured the height of a cylinder that resulted from different durations of photoexposure at J_e~33 mW·cm^{-2} of 405 nm light and plotted these working curves in Supplementary Figure 6. We can then fit this data to Supplemtary Equation (1):

$$C_d = A \log \frac{H_{e,0}}{H_{e,gelation}} = A\ (\log H_{e,0})\ - A \log H_{\mathrm{e,gelation}} \qquad \text{Supplementary Equation (1)}$$

Where A is a constant that captures the absorptivity of the resin, $H_{e,0}$ is the photodosage at the resin's interface and $H_{e,gelation}$ is the critical photodosage for gelation. With these working curves and an understanding of the irradiant density of our printer's light source, we can extrapolate the required exposure time to cure the resin one-layer height. In bottom-up SLA, exposure times below this value prevents adhering to previously printed layers whereas exposure times that exceed

this value cause cure through or a z-resolution that is worse than the nominal layer height. In practice, the z-resolution can be easily improved by the incorporation of non-reactive, highly absorptive dyes. We demonstrate the efficacy of this strategy by incorporating 0.1 g/mL of Sudan I into 64%MM10T to decrease the slope of the working curve. However, adding dyes to reduce layer height results in longer print times as the number of photoexposures increase.

To measure x-y resolution, we printed standard test geometries that contained regularly spaced square protrusions and cylindrical holes of varying size (250 – 2000 microns). We then directly measured the dimensions of these objects using optical microscopy. As shown in Supplementary Figure 6, we successfully (± 10% of target dimension) fabricated positive features down to approximately 250 microns using 70% MM10T for 23s full exposure. We also note that standard deviation for these features aligns closely with the nominal pixel resolution of the printer. Smaller objects may photopolymerize but fracture during the printing process.

For negative features, we can reliably print features within 10% of the target dimension down to 1000 microns. We propose that for smaller holes, the resin becomes entrapped within these wells for multiple photoexposures. Thus, partially reacted species are not sufficiently removed between build layers and the photodosage required for gelation is effectively lowered in subsequent steps. This theory is supported by the observation that the cylindrical holes become rounded with successive exposures; the most recently printed layer possess the highest resolution. We suggest venting negative features or other means of removing these partially reacted species (i.e, a prolonged wait time between successive exposures) to improve negative feature resolution.

<u>Shrinkage Post-Print:</u> Polymerization can often result in a change in volume, which threatens 3D printed resolution. We tracked the shape change during polymerization by using photo-rheology

(see above). During these experiments, the force between plates is measured and the instrument can actively adjust the gap height to maintain an axial force of 0.0 ± 0.1 N. By tracking this change in the gap height, we observed a 0.69 ± 0.02% (maximum value to minimum value, N = 3) for the pure thiol-ene material upon illumination (H_e ≈1600mJ·cm^{-2}). For the 82%MM10T resin, we observed a 0.11 ± 0.06% (maximum value to minimum value, N = 3) shrinkage with photoexposure to create the green-body and a 1.21% ± 0.08% total shrinkage when fully cured (18h RT, 2h 80°C). Additional thermal processing or photoexposure did not produce a perceptible change in volume (<0.01%) suggesting that both reactions are complete. For printed parts that require tight tolerances, this reduction in volume can be considered.

References

1. Wallin, T. J. *et al.* Click chemistry stereolithography for soft robots that self-heal. *J. Mater. Chem. B* **5**, 6249–6255 (2017).
2. Liu, Q., Nian, G., Yang, C., Qu, S. & Suo, Z. Bonding dissimilar polymer networks in various manufacturing processes. *Nat. Commun.* 1–11 doi:10.1038/s41467-018-03269-x
3. Song, J. & Van Ooij, W. J. Bonding and corrosion protection mechanisms of γ-APS and BTSE silane films on aluminum substrates. *J. Adhes. Sci. Technol.* **17**, 2191–2221 (2003).
4. Bennett, J. Measuring UV curing parameters of commercial photopolymers used in additive manufacturing. *Addit. Manuf.* **18**, 203–212 (2017).

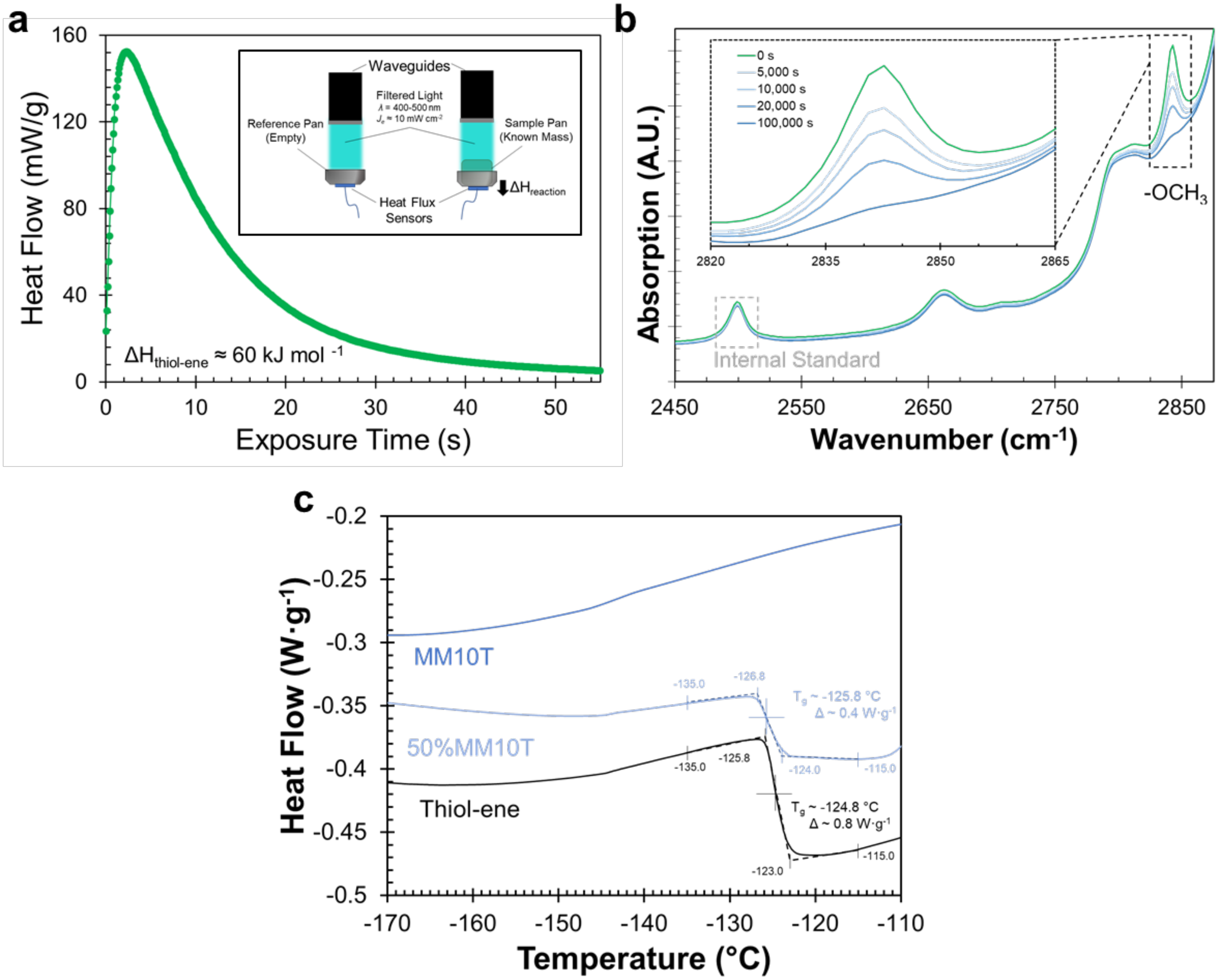

Supplementary Figure 1: Photo-Differential Scanning Calorimetry and Fourier Transform Infra-Red Spectroscopy of 82%MM10T. **a,** The normalized heat flow under photo-exposure for 82%MM10T. The inset portrays a schematic overview of the photo-DSC experiment. The exotherm shown is the average of N=3 runs. **b,** In-situ FTIR of the 82%MM10T material prior to (t = 0, green line) and after photo-exposure as a function of time (t = 5,000, 10,000, 20,000, and 100,000s as represented by the increasingly darker shades of blue). Inset emphasizes the change in area of the alkoxy (-OCH_3) peak. **c,** The normalized heat flow of fully cured Thiol-ene (black), 50%MM10T (light blue), and MM10T (dark blue) materials with their corresponding glass transitions (N=3).

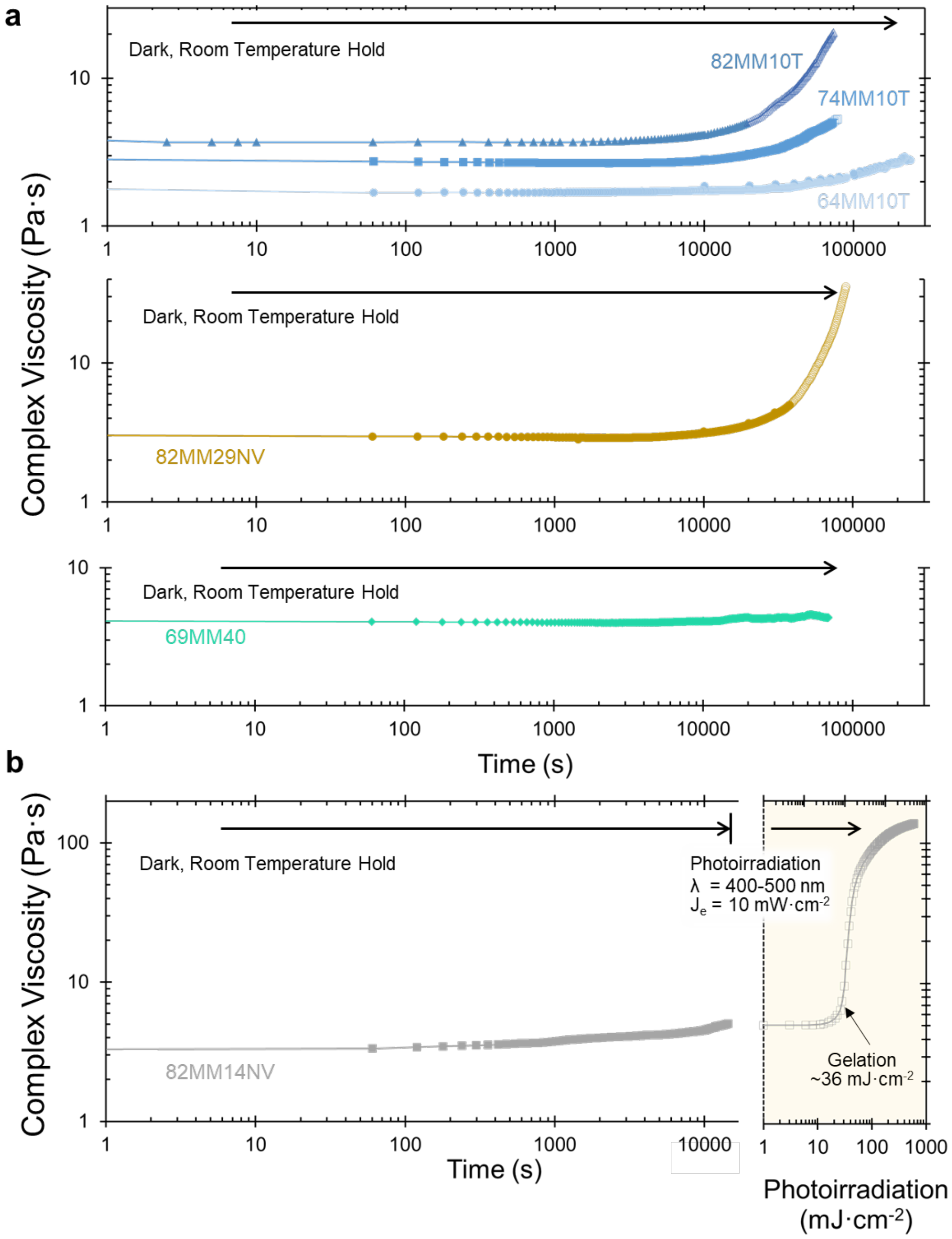

Supplementary Figure 2: Pot-life of Double Network Silicones. **a,** The time evolution of dark viscosity for representative SilDN materials. Open symbols denote points that exceed our viscosity threshold (5 Pa·s). An increasing thiol-ene content increases the pot-life of the materials. **b,** The time evolution of dark viscosity for 82%MM14NV. The material exceeds our viscosity threshold (5 Pa·s) after approximately 4 hours. After this period, we immediately begin photo-exposure to demonstrate that the material still cures in response to light. This behavior is consistent with orthogonal reactions between the thiol-ene and condensation networks.

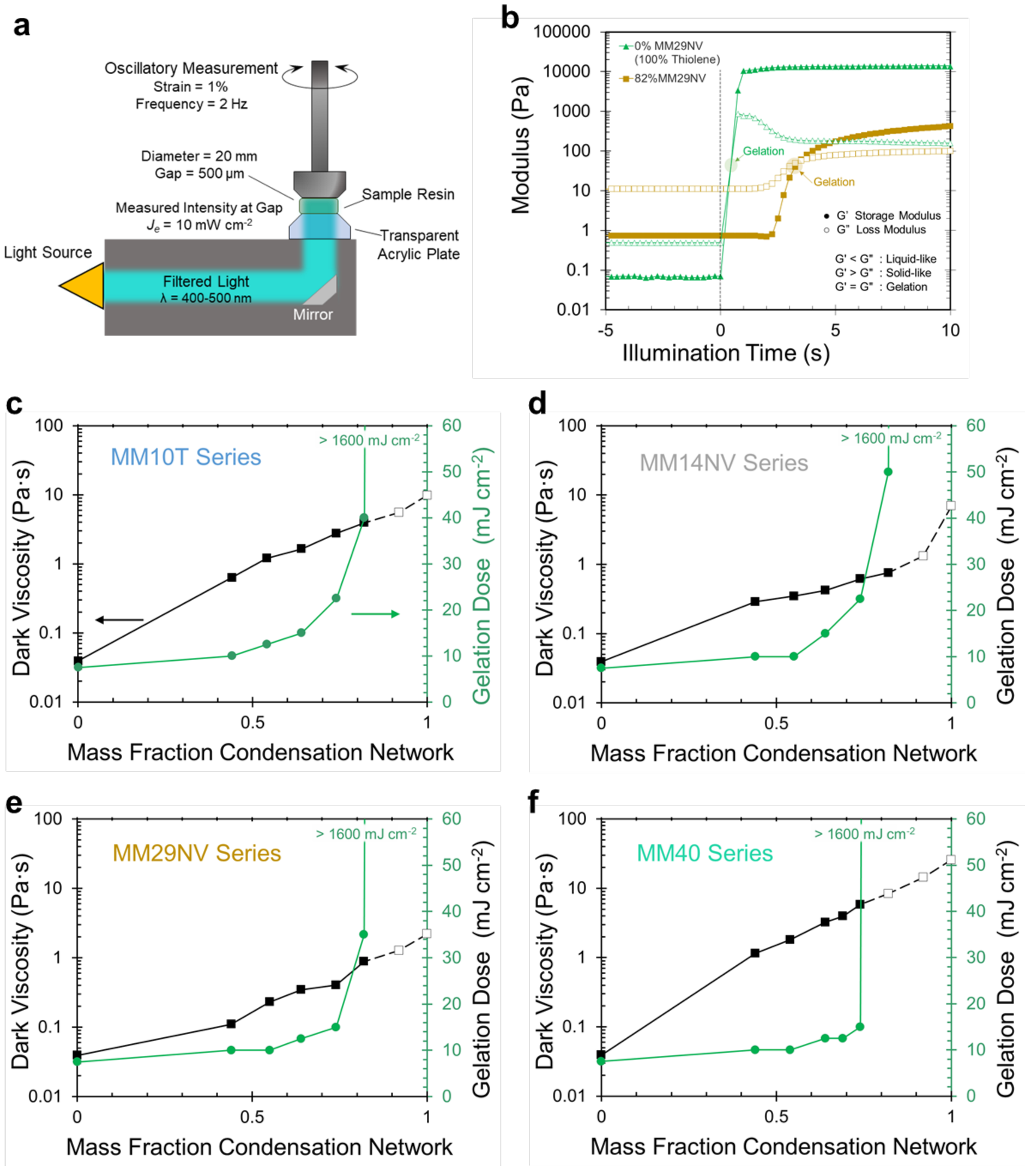

Supplementary Figure 3: Photorheology of SilDNs. **a,** A schematic overview of the photorheology experiment. **b,** Representative results (square symbols) that show the inferred gel dosage from the crossover in storage (colored symbols) and loss moduli (white symbols). Note the rapid plateau in moduli at low photo-dosages consistent with thiol-ene click chemistry (circle symbols). **c-f,** Gelation-dose and dark viscosity for the different SilDN material families: MM10T series (**c**), MM14NV series (**d**), MM29NV series (**e**), and MM40 series (**f**). Open circles denote blends that possess a photo-dose greater than 1600 mJ·cm^{-2} or a viscosity above 5 Pa·s, our thresholds for SLA printing. Each point is the average of $N = 3$ runs.

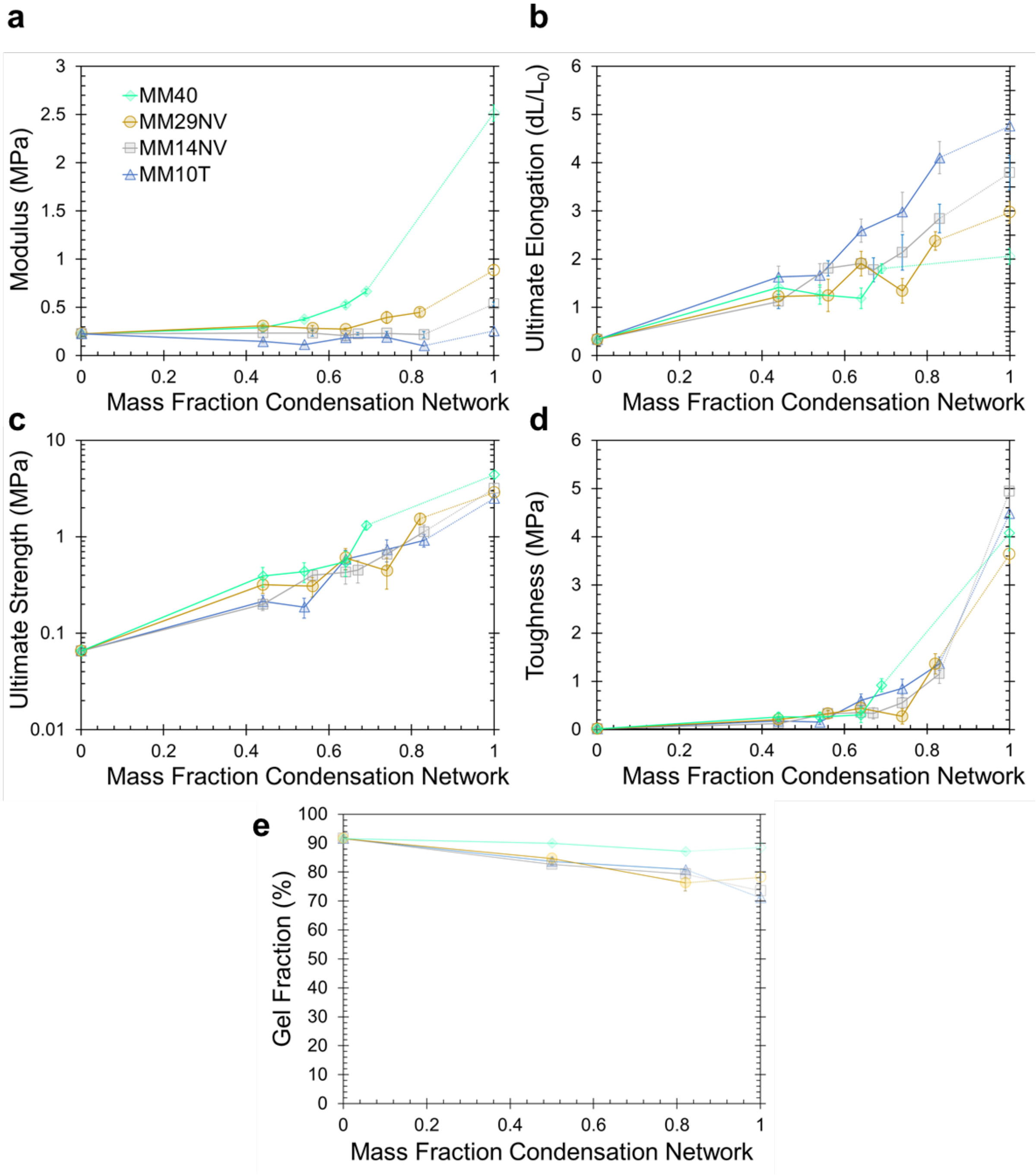

Supplementary Figure 4: Mechanical Performance of SilDNs. **a-d**, Summarized tensile data for the varying compositions of SilDNs including: the Young's Modulus at 100% strain (**a**), the Ultimate Elongation (**b**), the Ultimate Strength (**c**), and the Tensile Toughness (**d**). Open symbols denote blends that fail to meet our photo-dose (>1600 mJ·cm^{-2}) or viscosity (5 Pa·s) thresholds. Each point is the average of $N \geq 7$ coupons. **e,** The gel fraction, or amount of unextractable polymer, for varying blends ($N \geq 3$). For all figures, error bars represent standard deviation in the data.

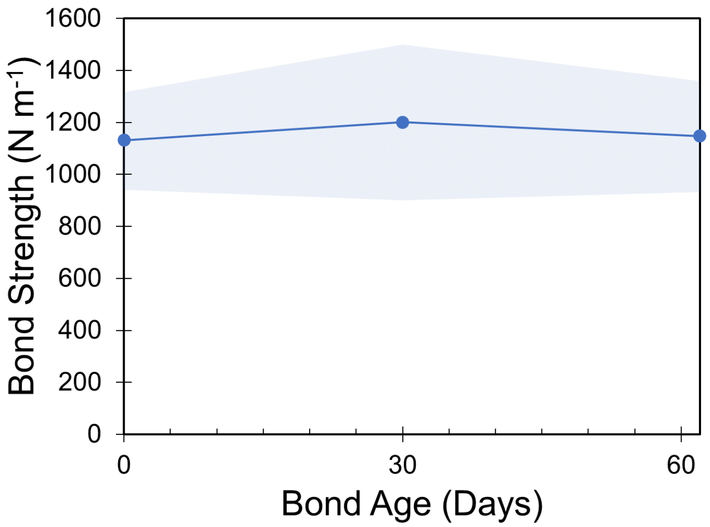

Supplementary Figure 5: Aging of SilDN Bonds. Self-bonded 82%MM10T held at ambient conditions show little change in bond strength over 60 days. All samples failed cohesively (N >7 samples, shaded regions represent standard deviation).

a

Negative Features

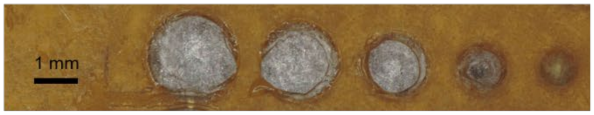

Target Diameter (μm)	Measured Diameter (μm) N ≥ 3
2000	2239 ± 158
1750	1920 ± 116
1500	1550 ± 135
1250	1311 ± 136
1000	980 ± 140

b

Positive Features

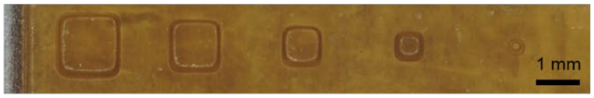

Target Length (μm)	Measured Length (μm) N ≥ 6
1250	1223 ± 65
1000	993 ± 46
750	710 ± 56
500	418 ± 57
250	226 ± 58

c

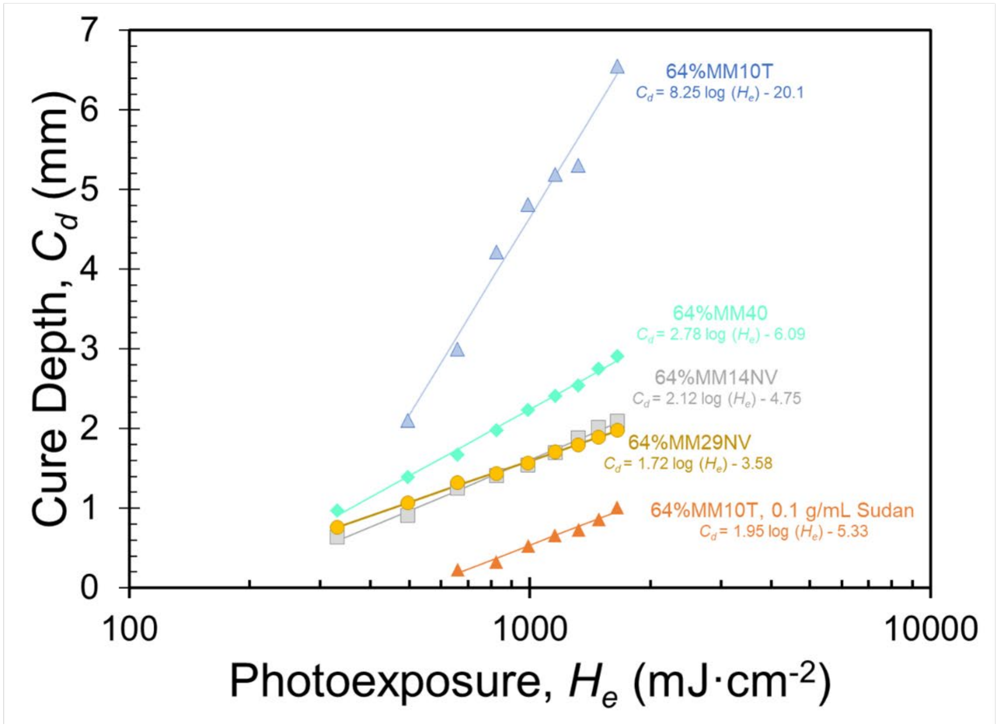

Supplementary Figure 6: Resolution of SilDNs. **a**, Negative features (holes) with varying diameters are printed within a 70%MM10T body and measured via microscopy (N > 3 printed objects). **b,** Positive features (square pegs) are printed on top of a 70%MM10T body and measured via microscopy (N > 3 printed objects, each measured vertically and horizontally). **c,** The working curve (cure depth v. log photoexposure) for varying SilDN compositions. The best fit for each data is shown.
