# Supplementary material for: 3D printable tough silicone double networks
Source: Nat Commun. 2020 Aug 10;11:4000. doi: 10.1038/s41467-020-17816-y (PMC7417997; doi:10.1038/s41467-020-17816-y)
Supplement: Supplementary file 3 — Description of Additional Supplementary Files [file 41467_2020_17816_MOESM3_ESM.pdf]

Description of Additional Supplementary Files

File Name: Supplementary Movie 1
Description: Stereolithography Printing with SilDNs

File Name: Supplementary Movie 2
Description: 3D Printed Surgical Simulator

File Name: Supplementary Movie 3
Description: Representative 90° Peel Tests

File Name: Supplementary Movie 4
Description: Steep and Shallow Stiffness Gradients from SilDNs

File Name: Supplementary Movie 5
Description: 3D Printed Interlocking SilDN Assemblies

File Name: Supplementary Movie 6
Description: Orthotic Glove with Dexterous Manipulation

File Name: Supplementary Movie 7
Description: Cyclic Donning and Doffing of Orthotic Glove 10 Months after Assembly

File Name: Supplementary Movie 8
Description: Bellows Actuator Attached to Flexible PCB
