# Supplementary material for: 3D printable tough silicone double networks
Source: Nat Commun. 2020 Aug 10;11:4000. doi: 10.1038/s41467-020-17816-y (PMC7417997; doi:10.1038/s41467-020-17816-y)
Supplement: Supplementary file 2 — Peer Review File [file 41467_2020_17816_MOESM2_ESM.pdf]

Editorial Note: This manuscript has been previously reviewed at another journal that is not operating a transparent peer review scheme. This document only contains reviewer comments and rebuttal letters for versions considered at Nature Communications.
